# A Stable Core–Shell Si@SiO_x_/C Anode Produced *via* the Spray and Pyrolysis Method for Lithium-Ion Batteries

**DOI:** 10.3389/fchem.2022.857036

**Published:** 2022-03-09

**Authors:** Xuelei Li, Wenbo Zhang, Xiaohu Wang, Wanming Teng, Ding Nan, Junhui Dong, Liang Bai, Jun Liu

**Affiliations:** ^1^ School of Materials Science and Engineering, Inner Mongolia University of Technology, Hohhot, China; ^2^ Inner Mongolia Key Laboratory of Graphite and Graphene for Energy Storage and Coating, Hohhot, China; ^3^ College of Chemistry and Chemical Engineering, Inner Mongolia University, Hohhot, China

**Keywords:** lithium-ion batteries, spray and pyrolysis methods, Si@SiOx/C anode, core–shell structure, excellent discharge capacity

## Abstract

In the critical situation of energy shortage and environmental problems, Si has been regarded as one of the most potential anode materials for next-generation lithium-ion batteries as a result of the relatively low delithiation potential and the eminent specific capacity. However, a Si anode is subjected to the huge volume expansion–contraction in the charging–discharging process, which can touch off pulverization of the bulk particles and worsens the cycle life. Herein, to reduce the volume change and improve the electrochemical performance, a novel Si@SiO_x_/C anode with a core–shell structure is designed by spray and pyrolysis methods. The SiO_x_/C shell not only ensures the structure stability and proves the high electrical conductivity but also prevents the penetration of electrolytes, so as to avoid the repetitive decomposition of electrolytes on the surface of Si particle. As expected, Si@SiO_x_/C anode maintains the excellent discharge capacity of 1,333 mAh g^−1^ after 100 cycles at a current density of 100 mA g^−1^. Even if the current density reaches up to 2,000 mA g^−1^, the capacity can still be maintained at 1,173 mAh g^−1^. This work paves an effective way to develop Si-based anodes for high-energy density lithium-ion batteries.

## Introduction

The lithium-ion battery is currently one of the high-performance rechargeable batteries in energy storage field, which has been commercialized and used in the portable electronic markets, renewable energy applications, and large-scale energy storage systems (Li et al., 2018; Lu et al., 2018; Lyu et al., 2020; Zhang et al., 2020). With the increasing energy/power density requirements and environmental problems in practical applications, it is critical to further improve electrochemical performances of the lithium-ion battery and reduce its price. Currently, graphite is still the most commonly used anode material for lithium-ion batteries, due to the advantages of high initial Coulombic efficiency, long life, non-toxicity, and low cost compared with other candidate materials (Yoshio et al., 2006; Khomenko et al., 2007; Yabuuchi et al., 2011; Yan et al., 2016). Nevertheless, graphite fails to satisfy the increasing energy density requirements as a result of the limited discharging capacity (the theoretical value of LiC_6_ = 372 mAh g^−1^). In recent years, Si is regarded as one of the most potential anode materials for the next-generation lithium-ion batteries in virtue of the eminent specific capacity (∼4,200 mAh g^−1^) and low delithiation potential (∼0.4 V vs. Li^+^/Li) (Lee et al., 2018; Nava et al., 2019; Zhou et al., 2020). Nevertheless, the extremely huge volume expansion/contraction (∼300%) in the lithiation/delithiation process has impeded the application of Si anode, which leads to the pulverization of bulk particles and deteriorates the long-term cycle life (Chan et al., 2008; Liu et al., 2014; Li et al., 2016; Cook et al., 2017).

In order to solve the aforementioned problems, great efforts have been made to suppress the volume expansion–contraction of Si anode and improve the structure stability, such as constructing porous nanostructure, nanosizing Si particles, using Si-based alloy, and coating a buffer layer (Lai et al., 2012; Lu et al., 2015; Woltornist et al., 2017; Han et al., 2020). Specifically, constructing a stable coating layer on the Si surface using carbon materials is a universal method to prolong the cycle life of the Si anode because using carbon as the coating layer can not only prevent the aggregation of Si nanoparticles and improve its conductivity but also can release the mechanical stress (Wu and Cui 2012; Wu et al., 2012; Liu et al., 2014; Horowitz et al., 2018). In particular, various Si/C nanocomposites with a core–shell structure using Si as the core and carbon as the shell are reported to show the high capacity and stable cycle performance in lithium-ion batteries (Kim et al., 2016; Nava et al., 2019; Zhou et al., 2019). In addition, to further enhance the structure stability of the Si/C anode during the cycling process, SiO_x_ is used as an intermediate layer between carbon and Si, which acts not only as a rigid buffer layer to limit Si particle expansion but also as a cohering layer to strengthen the interfacial cementation between the carbon shell and Si core (Jiang et al., 2016). However, shortages and problems including low production efficiency and incomplete coating for the preparation method are non-negligible. Therefore, it is necessary to advance a reasonable method that can realize rapid preparation and uniform coating for the Si anode.

The spray method is a stable and efficient method for preparing materials with nanostructures (Lai et al., 2013; Wang et al., 2016; Li et al., 2017; Zhang et al., 2017). The key of spray is to spray materials from the granulation nozzle, in which the target particles usually require to be atomized by high-pressure gas compression or high-pressure liquid. The ejected fog needs to be quickly dried by high temperature or hot air to form an anode material with a coating layer. However, nano Si is easy to combine with oxygen to form silica at high temperature. Therefore, inert protection gas such as nitrogen and argon needs to be used in the spray drying process, which increases the cost of the preparation process and is not conducive to large-scale preparation. Polymethylmethacrylate (PMMA) is often used as a carbon precursor to coat the surface of Si (Jiang et al., 2016). Besides, PMMA is also insoluble in water and will solidify once brought into contact with water. Considering these advantages of the spray method and the characteristics of PMMA in water, it is probably an effective strategy to spray the mixture solution of the target material (such as Si) and PMMA into water to achieve the complete coating, which does not require drying at high temperature or the inert gas protection.

Based on the previous analysis, a core–shell-structured Si@SiO_X_/C anode material with Si as the core and SiO_X_/C as the shell is prepared *via* the spray and pyrolysis methods. The PMMA polymer is used as both the oxygen and partial carbon source of the shell, PDA is used as a major carbon source of the shell, and nano-sized Si is used as the Si source. Due to the instructive buffering effect of the SiO_x_ interlayer and the complete carbon coating layer, the obtained Si@SiO_X_/C anode presents a high specific capacity of 1,333 mAh g^−1^ after 100 cycles at 100 mA g^−1^ and excellent rate capability. This work exploits a practicable solution for developing excellent performance Si anodes for Li-ion battery application.

## Experimental Section

### Material Preparation

#### Design of Spray Equipment

In order to approach the actual production, the equipment, that can already be mass-produced and used on the market, is selected as a basis of design. The spray nozzle (Shanghai Ou Meng OM-1500A spray dryer) is chosen to make the equipment. The nozzle of this equipment is an air atomizing nozzle, and the diameter of the prepared particles can be controlled below 2 μm, which is suitable for preparing carbon-coated Si anode materials. The peristaltic pump is mainly composed of three parts: 1) the control and driver parts, including electronic control circuit and motor, control the forward and reverse rotation of the motor; 2) the pump head of the peristaltic pump provides the peristaltic forward force for the liquid, which is mainly connected with the motor and the hose; and 3) an air compressor compresses gas, which provides mainly the stable high-pressure gas. Under the control of the peristaltic pump motor and the pump head, the organic liquid mixtures of Si and PMMA are quantitatively pumped into the air atomizing nozzle. Under the stable air pressure provided by the air compressor, the high-speed gas drives the organic solution to spray out at the nozzle to form micron-sized foggy droplets. These droplets are driven by the high-pressure gas into the rapidly stirred water to form PMMA-coated Si materials. Supplementary Figure S1 shows the schematic diagram of the spray equipment.

**FIGURE 1:**
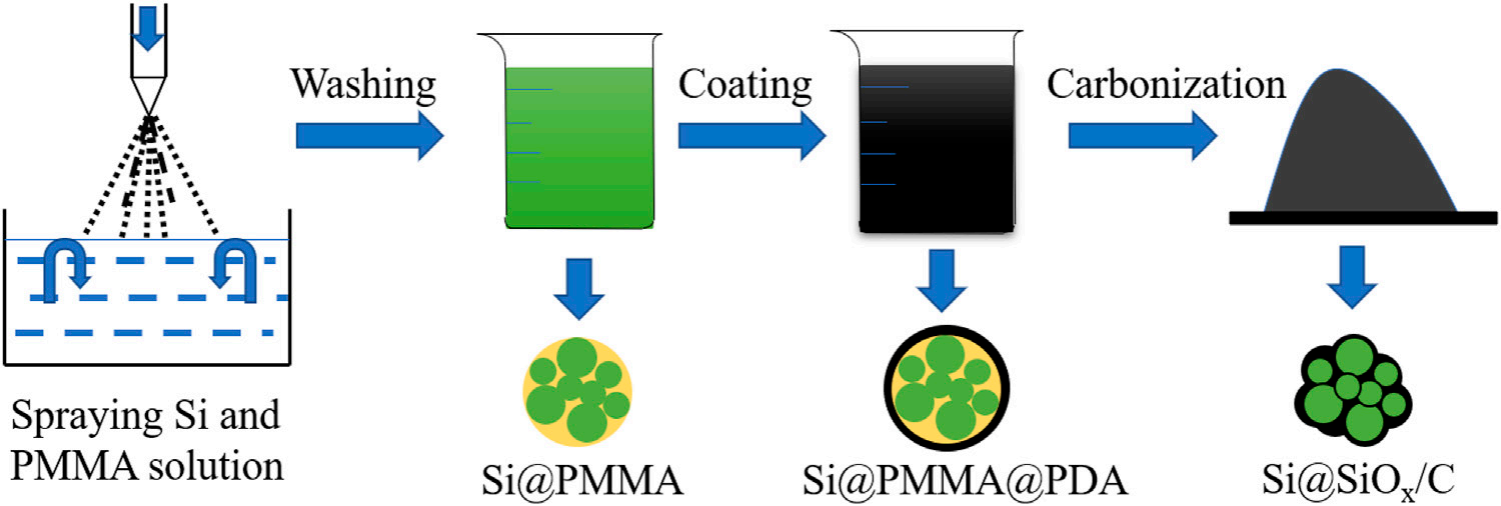
Flow diagram of preparation of Si@SiO_x_/C anode material by spray and pyrolysis methods.

Figure 1 illustrates the preparation process of the Si@SiO_x_/C material. The whole preparation process includes three steps: (a) preparing the PMMA-coated Si precursor (Si@PMMA) *via* the spray synthesis, (b) preparing the polydopamine (PDA)-coated Si@PMMA precursor (Si@PMMA@PDA) *via* the sol-gel method, and (c) carbonizing the Si@PMMA@PDA precursor to obtain the core–shell-structured Si@SiO_x_/C material.

#### Si@PMMA Preparation

In total, 0.5 g PMMA (analytical pure, Macklin) was added to 10 ml N,N-dimethylformamide (analytical pure, Macklin) solvent and stirred at 80°C for 2 h in a water bath to prepare the PMMA solution; 0.5 g Si particle (50 nm, Macklin) was added in 10 ml N,N-dimethylformamide (analytical pure, Macklin) solvent and dispersed by ultrasound for 2 h to prepare the Si solution. The as-prepared PMMA and Si solutions were mixed and then dispersed by ultrasound for 2 h as the PMMA/Si solutions. The temperature of distilled water in the water tank was controlled at about 30°C. The height from the nozzle to the water surface is about 30 cm, so that the air flow can just rush into the water without splashing. In the process of spraying particles, the air pressure at the output end of the air compressor is controlled at about 8 kPa, the pulsation pump is adjusted to 0.4 ml min^−1^, and the agitator is started to make the distilled water to form a vortex. After spraying, the solution was continuously stirred for 20 min to achieve uniformity. The sprayed solution was added to the distilled water and filtered through a vacuum suction filter to achieve the purpose of cleaning. It was repeatedly cleaned with the distilled water for 3 times to obtain Si@PMMA with gelation state.

#### Si@PMMA@PDA Preparation

The washed Si@PMMA was put into 250 ml distilled water and stirred rapidly for 6 h until a uniform suspension was formed. 0.3 g trihydroxymethylaminomethane was added into the uniform suspension and stirred for 2 h. Subsequently, 0.5 g polydopamine was added into the solution and stirred rapidly for 24 h to form black Si@PMMA@PDA solution after the pH value was adjusted to 8.5 through trimethylolaminomethane. The Si@PMMA@PDA solution was added into distilled water and filtered through a vacuum suction filter for cleaning. It is repeatedly cleaned with the distilled water 3 times to obtain the gelation state Si@PMMA@PDA. The obtained gelation state Si@PMMA@PDA was dried at 60°C for 24 h to obtain the Si@PMMA@PDA precursor.

#### Si@SiOx/C Preparation

The Si@PMMA@PDA precursor was ground and then sintered at 850°C for 1 h under flowing argon gas to obtain the core–shell-structured Si@SiO_x_/C material. According to the same route above, the mass of PMMA was changed from 1.0 to 1.5 g, and the obtained materials were abbreviated as Si@SiO_x_/C-2 and Si@SiO_x_/C-3, respectively.

### Materials Characterization

The structure of Si, Si@SiO_x_/C, Si@SiO_x_/C-2, and Si@SiO_x_/C-3 materials was detected by using X-ray diffraction (XRD, Rigaku D/MAX-2500, Japan) in the range from 10 to 80° with a step of 0.02°, using Cu-Kα radiation. The microstructure of the Si@SiO_x_/C material was explored by scanning electron microscopy (SEM, MERLIN Compact ZEISS Germany). The structure feature of the Si@SiO_x_/C material was analyzed by transmission electron microscopy (TEM, JEOL, JEM-2010-FEF) with energy-dispersive spectroscopy (EDS).

### Electrochemical Tests

The active material (Si, Si@SiO_x_/C, Si@SiO_x_/C-2 or Si@SiO_x_/C-3), carbon black (Super P), and sodium carboxymethyl cellulose (CMC) binder were mixed with distilled water to form a slurry on the basis of the weight ratio of 6:2:2. Then, the slurry was coated on copper foil with a thickness of 20 μm and immediately dried at 80°C in vacuum for 12 h to evaporate the distilled water. A Li disk and a porous polypropylene membrane were used as the counter electrode and the separator, respectively. 1 M LiF_6_PHO was dissolved in a mixture containing ethylene carbonate (EC), dimethyl carbonate (DMC), ethyl methyl carbonate (EMC), and 20% vinyl fluorocarbonate (FEC) as the electrolyte (EC: DMC: EMC = 1:1:1, by volume, Shinestar Battery Materials Co., Ltd., China). The mass loading of the active material was ∼ 0.8 mg cm^−2^ (0.628 mg). Sodium carboxymethyl cellulose was used as the adhesive, and the ultrapure water was used as the solvent. Coin-type half cells (2032-type) were used to evaluate the electrochemical performances of anodes. The cells were tested within a voltage range of 0.01–1.5 V (vs. Li/Li^+^) by a land battery testing system (Wuhan Kingnuo Electronics Co., Ltd., China) at 25°C. The assembled cells were first discharged-charged at 50 mA g^−1^ for three cycles and then carried out at 100 mA g^−1^ during subsequent tests. The rate performance of Si@SiO_x_/C anode was tested in the range of 50 mA g^−1^ to 2 A g^−1^. Cyclic voltammetry (CV) curves of Si@SiO_x_/C were obtained by a PMC electrochemical workstation. The potential range was 0.01–1.5 V, and the scan rate was 0.1 mV s^−1^.

## Results and Discussion

**FIGURE 2:**
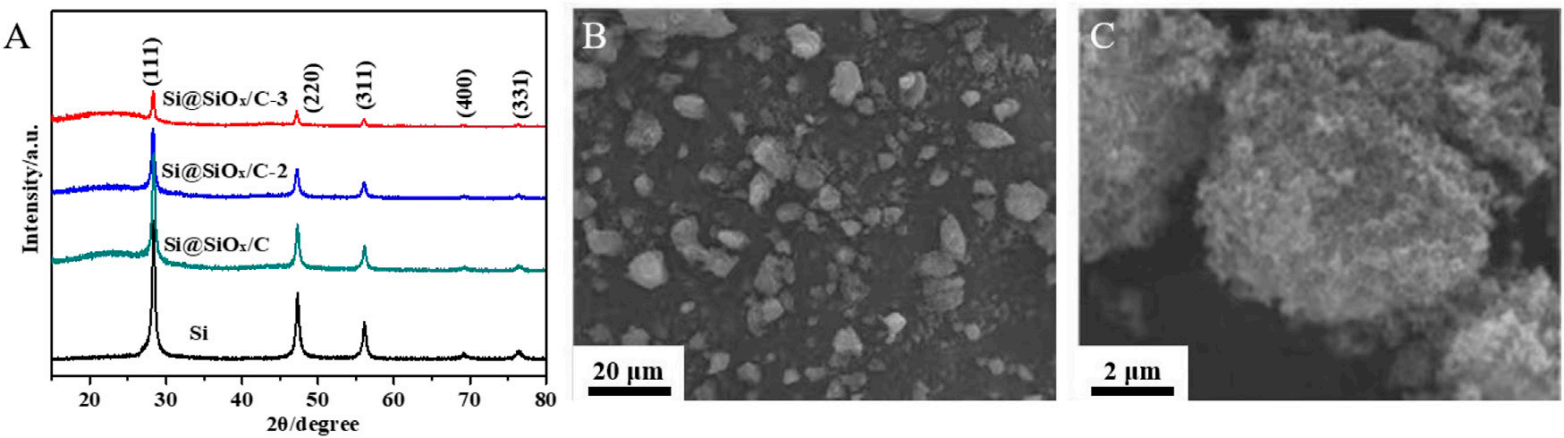
**(A)** XRD patterns of Si, Si@SiO_x_/C, Si@SiO_x_/C-2, and Si@SiO_x_/C-3 materials. SEM images of Si@SiO_x_/C material **(B)** at low multiples and **(C)** at high multiples.

Figure 2A shows the XRD patterns of Si, Si@SiO_x_/C, Si@SiO_x_/C-2, and Si@SiO_x_/C-3 materials. The sharp peaks of Si at about 2θ = 47.1°, 55.9°, 69.0°, and 76.3° are associated with (111), (220), (311), (400), and (331) crystal plane, respectively. However, the intensity of these peaks is gradually weakening from Si@SiO_x_/C, Si@SiO_x_/C-2 to Si@SiO_x_/C-3, indicating the thickness of carbon coating layer on the surface of Si is gradually increased. In addition, an obvious peak is displayed for Si@SiO_x_/C, Si@SiO_x_/C-2, Si@SiO_x_/C-3 at 2θ = 23°, indicating the obtained carbon is amorphous after pyrolyzing. During calcination of Si@PMMA@PDA precursor, Si will react inevitably with a small amount of O from PMMA to form SiO_x_, so the coating layer should be composed of a small amount of SiO_x_ and C layers. In addition, according to the report of Jiang et al., a SiO_x_ coating layer will be formed on the Si surface by a PMMA pyrolysis method (Jiang et al., 2016). However, the peak of SiO_x_ is negligible in an XRD pattern, indicating the content of SiO_x_ produced by the reaction is too low to be detected. SEM images of the Si@SiO_x_/C material are shown in Figures 2B,C that Si@SiO_x_/C particles are irregular with the size ranging from nanometer to micron, and each particle is an aggregation of many small crystalline solids.

**FIGURE 3:**
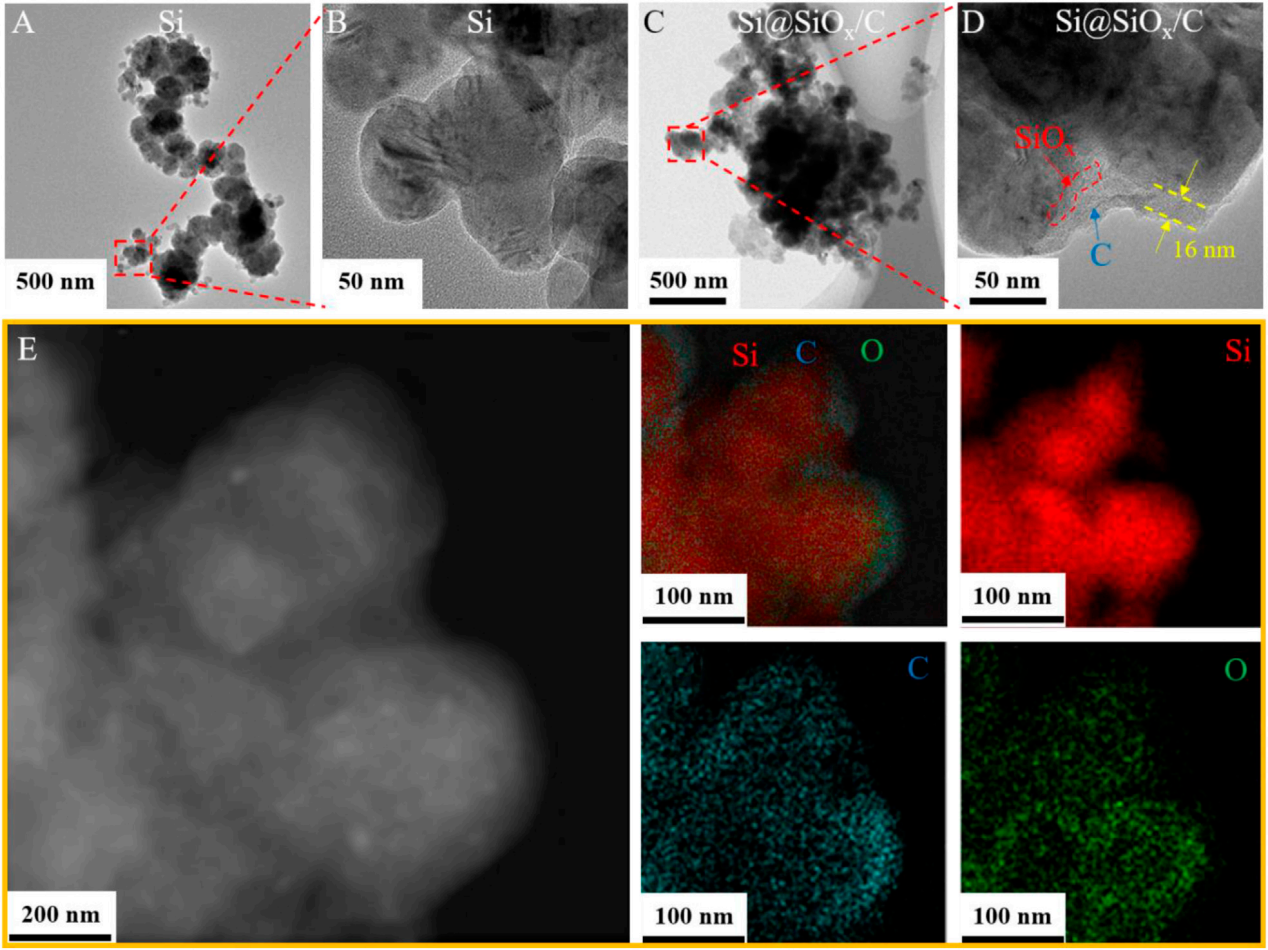
TEM images of **(A,B)** Si and **(C,D)** Si@SiO_x_/C materials. **(E)** TEM image and corresponding EDS mapping of Si, C, and O elements in a partial Si@SiO_x_/C particle.

Si, Si@PMMA, Si@PMMA@PDA, and Si@SiO_x_/C materials were tested by TEM to explore the variation of surficial coating layer in the preparation process. It can be clearly exhibited in Figures 3A,B that the surface of Si particle is smooth. TEM images of the Si@PMMA material shown in Supplementary Figure S2 indicate that PMMA is evenly coated on the surface of Si and the thickness of the coating layer is about 20 nm. Supplementary Figure S3 shows that the thickness of this coating layer increases up to 33 nm, illustrating that PDA is successfully coated on the surface of Si@PMMA. Furthermore, the core–shell structure can be distinctly discovered from the TEM image of a single Si@SiO_x_/C particle, as shown in Figures 3C,D. The Si core is perfectly covered with a uniform coating layer ∼16 nm thickness. During carbonization, Si reacts inevitably with a small amount of O from PMMA, so the coating layer should be composed of a small amount of SiO_x_ and C layers. Furthermore, the TEM image and corresponding EDS mapping of Si, C, and O elements in a partial Si@SiO_x_/C particle are shown in Figure 3E. Except for the agglomeration of Si element in the specimen, C and O elements distribute uniformly at the edge of the specimen, indicating the uniform coating of SiO_x_/C on the surface of Si particles.

**FIGURE 4:**
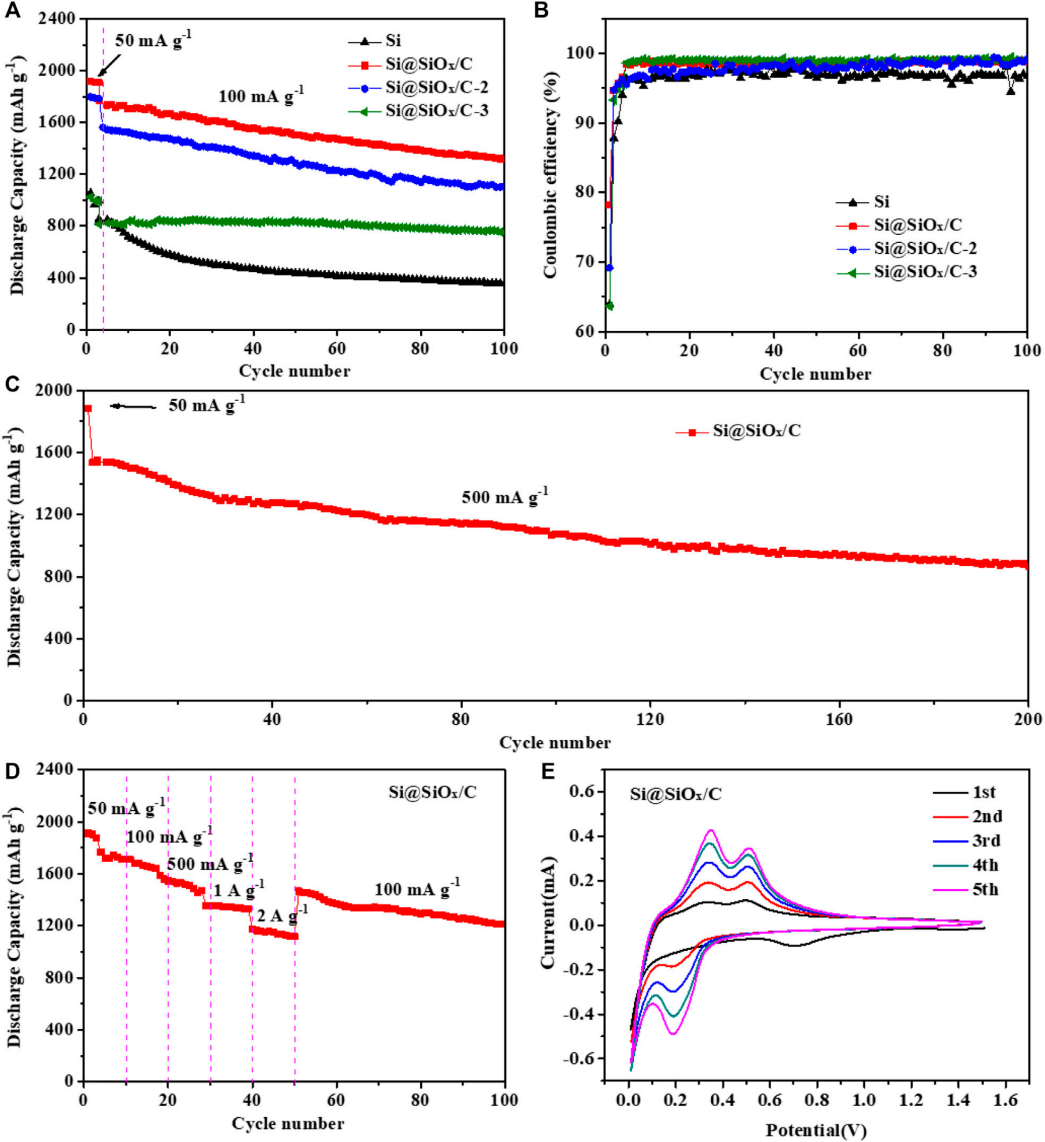
**(A)** Cycle performance curves and **(B)** corresponding Coulombic efficiency curves of Si, Si@SiO_x_/C, Si@SiO_x_/C-2 and Si@SiO_x_/C-3 anodes between 0.01 and 1.5 V at 100 mA g^−1^. **(C)** Cycle performance curves Si@SiO_x_/C anode at 500 mA g^−1^. **(D)** Rate performance curve of Si@SiO_x_/C anode and **(E)** CV curves of Si@SiO_x_/C anode from the 1st to 5th cycle at a scan rate of 0.1 mV s^−1^.

Figures 4A,B show the cycle performance profiles and the corresponding Coulombic efficiency curves of Si, Si@SiO_x_/C, Si@SiO_x_/C-2, and Si@SiO_x_/C-3 anodes at 100 mA g^−1^ after a three cycled activation process at 50 mA g^−1^. The initial discharging capacity of Si anode is 1053.7 mAh g^−1^. Si@SiO_x_/C anode displays the initial discharging capacity of 1919.7 mAh g^−1^. Remarkably, with the increase in the SiO_x_/C coating content, Si@SiO_x_/C-2 and Si@SiO_x_/C-3 anodes show the reduced initial discharging capacity. After 100 cycles, Si@SiO_x_/C anode can still maintain a discharging capacity of 1,333 mAh g^−1^. As a sharp contrast, the discharge capacity of Si anode rapidly decays to 600 mAh g^−1^ in the first 20 cycles, and a low capacity of 366 mAh g^−1^ is remained after 100 cycles. As for Si@SiO_x_/C, Si@SiO_x_/C-2, and Si@SiO_x_/C-3 anodes, the increase in coating content leads to the deceased discharging capacity and a more stable cycling performance (increasing the mass ratio of PMMA to Si). The reason can be ascribed to pyrolysis oxidization between O in PMMA and Si particles. Although the specific capacity is reduced, the structural stability and interfacial stability are increased (Jiang et al., 2016). Moreover, it can be found from Figure 4B that the initial Coulomb efficiency of Si, Si@SiO_x_/C, Si@SiO_x_/C-2, and Si@SiO_x_/C-3 anodes are 63.95, 78.24, 69.2, and 63.69%, respectively. According to the preparation process of anode materials, the increase in the thickness of SiO_x_/C layer is due to the increase in PMMA from Si@SiO_x_/C and Si@SiO_x_/C-2 to Si@SiO_x_/C-3. The increased PMMA leads to the increase in SiO_x_ ratio and the decrease in the carbon ratio in the SiO_x_/C coating layer. However, SiO_x_ is not as effective as carbon in improving the reversibility of lithium ions, which may be the reason for the decrease in Coulombic efficiency from Si@SiO_x_/C and Si@SiO_x_/C-2 and to Si@SiO_x_/C-3 anode. The loss of capacity is caused by the decomposition of electrolytes on the anode surface and the partial Li^+^ irreversible inserting in the Si lattice. In the subsequent cycles, the Coulombic efficiency of Si@SiO_x_/C, Si@SiO_x_/C-2, and Si@SiO_x_/C-3 anode maintained up to 98%, higher than that of Si (96%). This comparison illustrates that the progressive properties of Si@SiO_x_/C anode should be attributed to the protection role of the shell layer. To further confirm the protection role of the shell layer, the cycle performance of Si@SiO_x_/C anode at 500 mA g^−1^ is also tested. It can be shown in Figure 4C that Si@SiO_x_/C anode can still maintain a high discharging capacity of 866.1 mAh g^−1^ after 200 cycles. The aforementioned results explain that the SiO_x_/C shell not only ensures the structure stability but also impedes the permeation of electrolytes, thus preventing the repeated electrolyte reduction happening and achieving the high cycle performance.

Figure 4D shows the rate performance of Si@SiO_x_/C anode. As the current increases from 50 to 500 mA g^−1^, the capacity of Si@SiO_x_/C anode decreases slightly from 1910.7 to 1529.5 mAh g^−1^. Even at higher currents of 1,000 and 2,000 mA g^−1^, Si@SiO_x_/C anode can still deliver reversible capacities of 1352.6 and 1173.0 mAh g^−1^. Obviously, such a high-rate capability of Si@SiO_x_/C anode should benefit from the SiO_x_/C shell, which can not only remarkably improve the conductivity but also provide a fast Li^+^ transmission channel for internal Si alloying reactions. Figure 4E shows the CV curves of Si@SiO_x_/C anode at the scanning rate of 0.1 mV s^−1^ from the 1st to 5th cycle. A large, broad cathodic peak at 0.69 V could be attributed to the formation of the passivation interface layer. In the subsequent cathodic process, the redox peak at 0.19 V corresponds to the alloying reaction between Si and lithium (Si + xLi^+^ + xe^−^ → Li_x_Si) (Tian et al., 2021). In the first charge process, two anodic peaks at 0.33 and 0.50 V appear, which is in correlation with the dealloying of Li_x_Si. From the second cycle, the redox peaks become sharper and gradually tend to overlap, indicating excellent reversibility and stability of Si@SiO_x_/C anode.

## Conclusion

In summary, an effective strategy is designed to construct the core–shell Si@SiO_x_/C anode for lithium-ion batteries. XRD and EDS mapping clearly indicate the proportion of composition of the SiO_x_/C shell. The SEM image shows that the Si@SiO_x_/C material is the irregular aggregate of many small crystalline solids with their size ranging from nanometer to micron. TEM image demonstrates that the core–shell structure is successfully synthesized. Furthermore, the as-prepared Si@SiO_x_/C anode demonstrates the high discharging capacity, good rate capability, and cycle ability. The excellent properties of Si@SiO_x_/C anode should get benefitted from the unique core–shell structure, in which the SiO_x_/C shell not only ensures the structure stability and proves the high electrical conductivity but also hinders the permeation of electrolyte, thus refraining from the continual reduction of electrolytes on the surface of Si and realizing an excellent cycle performance. This work provides a promising strategy for the development of high-performance Si anode for lithium-ion batteries.

## Data Availability

The original contributions presented in the study are included in the article/Supplementary Material, further inquiries can be directed to the corresponding authors.

## Supplementary Material

Supplementary Figure S1 Schematic diagram of the spray equipment.

Supplementary Figure S2 TEM images of Si@PMMA materials.

Supplementary Figure S3 TEM images of Si@PMMA@PDA materials.
