# Supplementary material for: A Stable Core–Shell Si@SiOx/C Anode Produced via the Spray and Pyrolysis Method for Lithium-Ion Batteries
Source: Front Chem. 2022 Mar 9;10:857036. doi: 10.3389/fchem.2022.857036 (PMC8959764; doi:10.3389/fchem.2022.857036)
Supplement: Supplementary file 1 [file DataSheet1.docx]

**Supplementary Material**

**A stable core-shell** **Si@SiO_x_/C anode produced via** **the spray and pyrolysis method for lithium-ion batteries**

Xuelei Li^1,3^, Wenbo Zhang^1,3^, Xiaohu Wang^1,3^, Wanming Teng^1,3^, Ding Nan^1,2,3^*, Junhui Dong^1,3^, Liang Bai^1,3^, Jun Liu^1,3^*


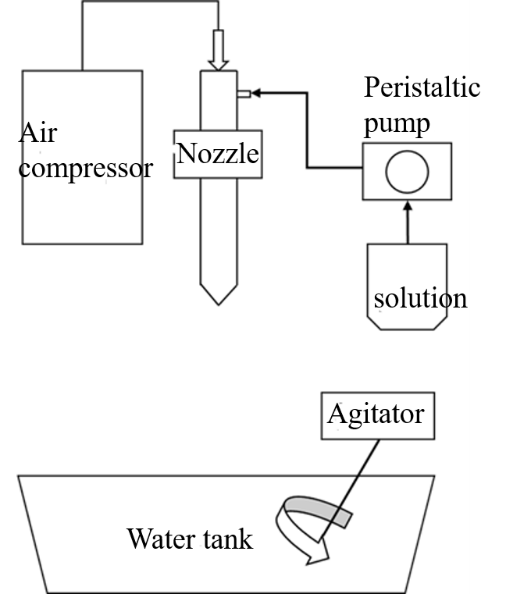


**FIGURE S1.** Schematic diagram of the spray equipment.

**
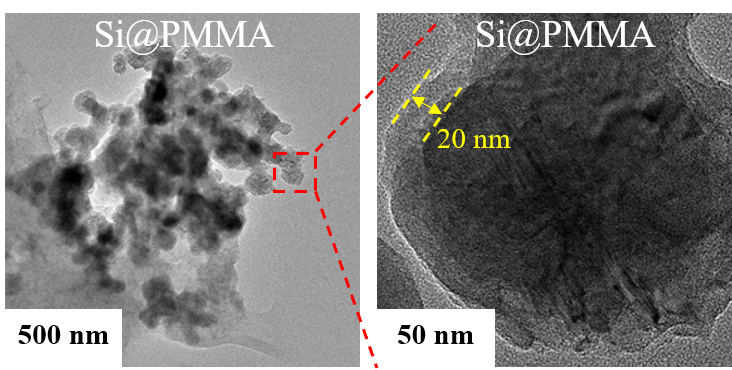
**

**FIGURE S2.** TEM images of  Si@PMMA materials.

**
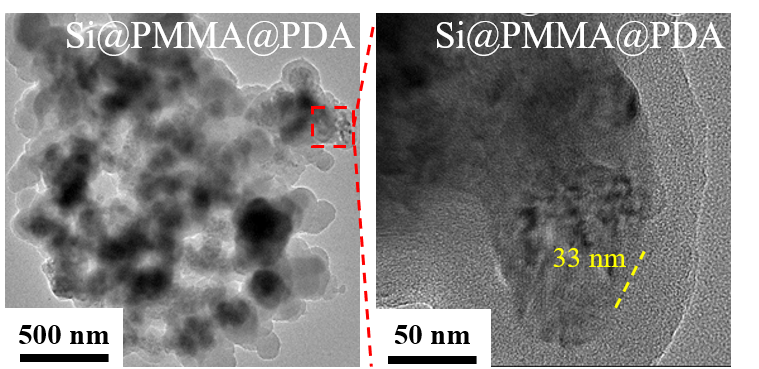
**

**FIGURE S3.** TEM images of Si@PMMA@PDA materials.
